# A phenomenological study of differentiated instruction experience in an Ethiopian middle school: The case of grade 7 students in Hawssa city, Ethiopia

**DOI:** 10.1371/journal.pone.0341025

**Published:** 2026-01-16

**Authors:** Fozia Temam Mohammed, Taye Gebremariam Olamo, Mesfin Abera Yemiru

**Affiliations:** College of Social Sciences and Humanities, Hawassa University, Hawassa, Ethiopia; National University of Malaysia Faculty of Education: Universiti Kebangsaan Malaysia Fakulti Pendidikan, MALAYSIA

## Abstract

This phenomenological study investigates middle school students’ and their English language teacher’s experience with differentiated instruction in a writing classroom. The aim was delving into the lived experiences of the students and the teacher to uncover insights as well as benefits and challenges encountered in writing instruction. The study was conducted at Stadium Elementary School in the city of Hawassa, Ethiopia. A total of 32 middle level students aged 13/14 and their English language teacher were participants of the study. Of these, six students and the teacher were subjects for the interview. Data were gathered through face-to-face interviews and observation of a classroom. Data was analyzed thematically. The findings of the study revealed that differentiated instruction provided students with tailored support, leading to enhanced motivation, engagement, and confidence in their writing abilities. As a challenge some students’ unfamiliarity and resistance to the approach emerged Moreover, the teacher’s experience revealed the demanding nature of the approach and need of collaboration from stakeholders. We conclude that, when the concerns raised by research participants are effectively addressed, the incorporation of differentiated instruction has strong potential to improve students’ writing outcomes and engagement, offering a practical approach for educators seeking to personalize learning and enhance writing quality.

## 1. Introduction

In the regular classroom it is inevitable to have students with various needs. The variation can range from physical characteristics, intelligence, perception, gender, ability and learning styles [1]. According to [2], there are three students’ characteristics teachers need to take into consideration while crafting curriculum and instruction: readiness (current knowledge, understanding and skill level of learners); interest (what a student enjoys learning about, thinking about and doing) and learning profile (preferred mode of learning, which in turn is influenced by learning style, intelligence preference, gender and culture). Students with these diverse characteristics are put in one classroom and are expected to learn with similar pace and support system with their classmates. This one size fits all approach to teaching has remained to be a challenge to education. To use Oliver’s words, an instructional approach which uses only one way of teaching to all students by considering students learn in a similar way harms the students’ growth [3].

Teachers can be more responsive to their learners’ individual differences by becoming aware of the differences among learners [4]. If educational practice is to progress teachers need to find a way to deal with diversity and the needs of individuals, while teaching a group of students (Visser, 1998) as cited in [3]. A proven educational approach, among others, that takes individual differences into consideration and tailor instruction to cater to the differences among learners is differentiated instruction (DI) [4–6] and [7]. [8] one of a leading expert in the field of DI stated the concept of accommodating learning differences as a way of thinking about the classroom with dual goals of honouring each student’s learning needs and maximizing each student’s learning capacity. Tomlinson also added that “a differentiated classroom provides different avenues to acquiring content, to processing or making sense of ideas, and to developing products so that each student can learn effectively” (p.1). According to this definition a teacher is expected to be active and continuously plan that considers and reflects understanding of student differences, and that accommodates the various ways that students learn.

Scholars in the field of DI have designed different models that emphasize and discuss different elements of education that need to be modified to meet the diverse needs of students. Tomlison forwarded a model that addresses differentiation from modification of four curricular elements: content, process, product and learning environment as a response to students’ characteristics of readiness level, interest and learner profile. [7] model explored DI from more concrete and comprehensive dimensions. By incorporating more elements to Tomlinson’s model these scholars introduced The Five Dimensions of Differentiated Instruction which encompasses teaching arrangements, the learning environment, teaching methods, support materials, and assessment. Another model by [6] identified five elements of education to be responsive for: content, instructional strategies, the classroom, products and the teacher.

These models highlight the importance of DI in tailoring instruction to meet learners’ diverse needs. Implementing DI can enhance student engagement, accommodate various learning styles, and improve educational outcomes. Research indicates that DI can increase students’ engagement and academic performance [9,10] and [11], motivation, and reduce stress [12], while also improving skills such as writing [13] and [14]. Studies by [15] and [16] confirm positive effects of DI on vocabulary and grammar achievement, respectively. It also brings positive impact on students’ perception and motivation [17]. According to [18] and [19] Students’ positive perception is said to facilitate learning.

While there are several benefits of DI, it is important to acknowledge that its demanding nature has also been highlighted by other researchers. Researchers emphasize the significant time, effort, and dedication required from teachers [20] and [5]. [21] tells us that time constraint remains the most significant challenge among the challenges of implementing DI to meet the needs of all students. Teachers fall short of time to assess students, to know their needs, interests and readiness levels and to design lessons as a response to existing differences. [22] listed lack of resources, time, support, knowledge and class size as barriers for DI implementation. Another challenge, highlighted by [21] is classroom management, coupled with the shift in teachers’ roles from knowledge dispenser to learning facilitator. Additionally, teachers’ readiness and willingness to adopt and implement new strategies within DI are key. Significance of contextual factors that can either facilitate or hinder DI implementation are also enumerated in previous studies of [23–25] and [26].

Implementing DI successfully demands teachers’ commitment to making this approach work in the classroom. Despite the challenges enumerated for the successful implementation of DI, teachers have positive outlook towards DI [27]. However, they need professional development opportunities for effectively implementing this approach in their day-to-day teaching practice [28]. [29] also mentioned teachers’ knowledge and training gap on how to implement DI, scarcity of school resources and conducive school environment, teachers’ low motivation and commitment, rigid curriculum, work load of teachers, absence of support from school administration and parental involvement, students’ poor background knowledge, and weak collaboration among staff members as hindering factors for DI implementation.

DI is a key focus area in Ethiopia’s education. While research supports its effectiveness in enhancing student engagement, motivation, and academic achievement, DI has not been extensively studied within the Ethiopian context, with only a few studies conducted to the knowledge of the current researchers. For instance, [30] explored teacher educators’ perceptions and experiences regarding DI at three Teacher Education Colleges in Ethiopia discovering a positive perception of DI among teacher educators for use in diverse classrooms. Likewise, [16] examined grade 12 students’ perceptions of the DI approach, revealing a similarly positive outlook towards this instructional strategy. Additional studies by [31] and [15] investigated primary school teachers’ perceptions of DI in two different contexts within Ethiopia, both yielding findings that indicated a positive perception of DI among teachers. However, the experiences of teachers and students with DI, particularly at the middle school level, remain largely unexamined. Furthermore, there is a need for more research on how teachers implement DI strategies in teaching writing skills and how students respond to these approaches. Addressing this gap is essential to better understand the practical challenges and successes in integrating DI into writing instruction at this level.

Therefore, this study aims to explore the insights of middle school students and their English language teacher regarding DI, as well as the enabling and challenging factors they encounter during its implementation.

### 1.1 Theoretical framework

The theoretical foundations of differentiated instruction (DI) are rooted in Vygotsky’s social constructivist theory and Gardner’s Multiple Intelligences (MI) theory. Vygotsky emphasizes social interaction, collaborative learning, and the Zone of Proximal Development (ZPD), which highlights the importance of identifying learners’ current abilities and providing targeted support to help them progress beyond their independent capacities, thus fostering student-centered learning [32] and [33]. Complementing this, Gardner’s MI theory asserts that students possess diverse kinds of intelligences—such as linguistic, logical-mathematical, musical, spatial, bodily-kinesthetic, interpersonal, intrapersonal, naturalist, and existential—that should be tapped through varied teaching strategies [34]. Recognizing multiple intelligences encourages educators to diversify instructional methods, ensuring that teaching caters to individual differences, enhances engagement, and promotes academic success by providing multiple avenues for students to access, process, and demonstrate their understanding, thereby making the classroom more inclusive and effective.

## 2. Objectives of the study

This phenomenological study investigates grade seven students’ and their teacher’s lived experiences and insights with DI. It focuses on investigating the enablers, challenges and benefits participants experienced while dealing with writing through DI.

### 2.1 Specific objectives

The specific objectives of this research attempted to achieve were:

1. exploring students’ reflection towards DI approach
2. investigating the teacher’s reflection towards DI while teaching writing through it.
3. exploring facilitators and barriers in dealing with writing through DI approach.

## 3. Materials and methods

### 3.1 Study design and research approach

This study employs a qualitative phenomenological study design which is grounded in an interpretative paradigm [35, p.8]. This approach is selected because it gives a chance to examine people’s experience with a phenomenon (DI approach in writing classroom in this work’s context) and their interpretation of it in this work’s context. Phenomenological research lends itself to inquiries that try to learn “…what has been experienced in terms of the phenomenon and what contexts affected or influenced those experiences” [36, p.472]. Qualitative research is also suitable to meet research participants in their natural settings [37, p4]. By employing qualitative research, this study allows the researchers to meet the students and their teacher in their natural setting and thereby examine, understand and interpret their experiences

### 3.2 Study design and research approach

This study employs a qualitative phenomenological study design which is grounded in an interpretative paradigm [35, p.8]. This approach is selected because it gives a chance to examine people’s experiences with a phenomenon (DI approach in writing classroom in this work’s context) and their interpretation of it in this work’s context. Phenomenological research lends itself to inquiries that try to learn “…what has been experienced in terms of the phenomenon and what contexts affected or influenced those experiences” [36, p.472]. Qualitative research is also suitable to meet research participants in their natural settings [37, p.4]. By employing qualitative research, this study allows the researchers to meet the students and their teacher in their natural setting and thereby examine, understand, and interpret their experiences.

### 3.3 Study setting

The setting of this study was in a public middle school found in the Sidama Regional State of Ethiopia. The school is found in Hawassa City Administration, the capital of the region which is 273 km away from Addis Ababa, Capital City of Ethiopia.

### 3.4 Study participants and sampling techniques

The study participants were grade 7 students in a primary middle school. Pseudonyms were used to refer to the teacher and the students for confidentiality purpose. Purposive sampling was employed to select the school. The school was selected for its proximity and its ongoing implementation of DI in teaching writing to students at the time the data was collected. There were four 7th-grade sections in the school; however, one particular section comprising 32 students was selected for this study due to the ongoing implementation of DI in this specific class. Participants in a phenomenological study are selected due to the fact that they have been through the experience (DI in this case) and they can share their experiences about it [36]. The school principal and the teacher were collaborative to provide the needed data.

Students in grade 7 are generally between 12 and 13 years old. This age group is chosen because it is a critical time for students to prepare to learn English, not only as an academic subject but also as a means of comprehending and studying other subjects throughout their academic career, starting from grade nine and continuing into higher education [38,pp.30–31] The study involved 32 participants (12 males, 20 females), including 1 English language teacher and conducted during the second semester of the academic year 2024.

This research was carried out in line with ethical guidelines and received approval from the Institutional Review Board (IRB) at Hawassa University’s College of Social Sciences and Humanities. We secured an official ethical approval certificate to uphold the rights of the participants. We communicated openly with the school leadership about the objectives of the study, ensuring that participants were fully aware that their involvement was voluntary and that they had the right to withdraw at any point. These actions helped to build trust and ensure adherence to ethical practices. Participants were selected on 20 April, 2024. Assent was obtained from them, and written consent was subsequently sent to their parents or guardians on the same date. The consent forms were collected back on 21 and 22 April, 2024. Since the participants were minors, securing parental or guardian consent was an essential part of the ethical protocol that was needed to adhere to. The classroom observation was conducted during the month of May 2024, followed by interviews with the teacher and selected students. The same procedure was followed to secure consent from the participating teacher.

After receiving site permissions, the researchers met with the English language teacher to discuss the study’s goals and the importance of using pseudonyms to maintain anonymity. Informed consent was obtained from both the teacher and the parents of the students, in accordance with ethical standards due to the participants’ age. Additionally, we sought assent from the minors, providing them with appropriate information about the study and reiterating their right to withdraw at any time. This approach ensured ethical standards were met and demonstrated respect for the autonomy of the students.

### 3.5 Data gathering instruments

To gather the important information from the research participants, semi-structured interview and classroom observation were used.

#### 3.5.1. Observation.

Classroom observation was one of the data gathering instruments that was used in this study because it allows the researchers to observe live classroom practices. It is useful data collection tool for it gives live data. The researchers designed their own observation checklist from the literature they have consulted for the purpose of this work which helped them achieve the objective of the research. The checklist is designed based on the reviewed literature on DI, its elements and strategies used to implement it in the classroom. The observation checklist was presented in a tabular format with specific parameters such as teaching methods, assessment techniques, student engagement, student behaviour, and grouping strategies. We emphasized that each parameter included space for observers to record their observations and notes, facilitating systematic and detailed documentation of classroom practices related to Differentiated Instruction.

The validity of this instrument is established by incorporating comments from experts in the field. To ensure the reliability of the observations, two observers independently visited the classroom with separate observation checklists, recorded their findings, and then discussed their results together to achieve consistency in interpretation.

In addition to this, the observation was done in eight rounds for 40 minutes duration each to increase the reliability of the result of the observation. The observation was carried out during the regular teaching-learning time. Together there were 8 observations of a classroom or observations of 320 minutes were conducted. It took 8 days to complete the observation during the second semester of 2024 academic year.

#### 3.5.2. Semi-structured interview.

A semi-structured interview was used as a data collection tool, allowing researchers the flexibility to modify and add questions as needed [39]. Semi-structured interview questions also do not restrict the views of the respondents by providing predesigned answers to questions as in closed ended questions [40]. Interview is also said to be the primary method of data collection in this research type [36] and [37]. For this purpose, an interview guide was developed, and researchers conducted one-on-one interviews with six students—two from each achievement level (high, average, low)—to understand their reflections on the teaching approach. The criteria for selection included the teacher’s judgment, which was based on ongoing assessments such as observations, one-on-one discussions, and reviews of students’ written extracts, combined with students’ previous semester writing performance. By integrating these assessment data, students were classified into three readiness levels for differentiated instruction. Six students were then selected from different performance groups to ensure a diverse representation of achievement levels and learning needs.

The students, were given pseudonyms as S1–S6, were addressed by these codes throughout the discussion to protect their identities. The students’ interviews focused on their overall reflections regarding the DI strategies employed, the level of support they received, as well as the advantages and disadvantages they encountered as a result.

The teacher’s reflection towards DI approach was also elicited via the interview. The teacher’s interview cantered on his experiences with DI, including challenges and enablers, as well as his perceptions and the suggestions he offered for incorporating DI into English Language Teaching (ELT) classrooms. Student interviews were tape-recorded for transcription and analysis, while the teacher’s responses were noted down as he did not consent to audio recording.

The interview questions were pilot-tested on non-sample individuals and reviewed by experts for quality. The interviews were conducted in the students’ local language to ensure the conversation was suitable for the interviewees’ language proficiency. The recorded data was transcribed, and subsequently, translated into.

### 3.6 Data collection procedures

After gaining an access to the research sites and making the purpose of the research clear to the participants, classroom observation was conducted first so that participants’ action cannot be influenced by the ideas reflected on the interview which was to come later. The observation was conducted based on the already designed and validated observation checklist which is also accompanied by field notes for further analysis. The observation results were consistent across all parameters in each session. For each of the eight observation sessions involving 32 participants, including the English writing teacher, the findings from the checklist observations and the field notes showed the same results for each aspect observed. In cases where discrepancies may have occurred, the observers conducted debriefing sessions to compare and discuss the data, ultimately determining the final observation results based on the consensus reached during these discussions.

Following the classroom observations, semi-structured interviews were conducted with both the teacher and students. This method allowed for a flexible yet focused approach to gathering insights, facilitating an exploration of participants’ perspectives and experiences within the classroom setting. The interviews with participants were scheduled to occur immediately after the completion of the instructional activities to capture their immediate reflections. Each interview lasted approximately 15–20 minutes, allowing enough time for open-ended questions and detailed responses. The interviews were conducted individually in a quiet, designated area to ensure confidentiality and comfort. Participants were informed about the purpose of the interview beforehand, and consent was obtained prior to the sessions. This approach aimed to gather in-depth insights into their perceptions of the teaching strategies, challenges faced, and perceived improvements in their writing skills.

### 3.7 The learning process

During the observation process, the teacher implemented DI primarily through content, process and learning environment differentiation, aligning with Tomlinson’s theory. The curriculum was modified to cater to students’ varying readiness levels. Specifically, the teacher employed flexible grouping strategies—forming homogeneous, heterogeneous, whole-class, and individual groups—to facilitate targeted instruction and foster peer support. Students were provided with different writing prompts based on their readiness level and they were also gaining different levels of scaffolding. Learning environment was differentiation was employed via arranging seating to facilitate collaboration, fostering a classroom culture that values diversity and effort.

To address diverse student needs, different learning materials and tasks were assigned. Struggling students received scaffolded activities such as word banks, sentence starters, and cohesive devices, alongside home reading tasks designed to fill gaps in their knowledge and skills. These scaffolding strategies provided additional support to ensure their active participation and progress. High-achieving students were given extension tasks aimed at deepening their understanding and encouraging higher-order thinking, which they often worked on collaboratively with struggling students to serve as peer tutors and facilitators. Students at the middle level worked on tasks tailored for their proficiency, with moderate support from both the teacher and peers.

In terms of teaching writing through group work, students collaborated to prepare outlines for paragraph writing. The process involved brainstorming ideas collectively, generating content, and structuring their paragraphs. This collaborative approach fostered peer learning and enabled students to learn from one another within a supportive environment.

Overall, the teacher’s differentiated instruction effectively accommodated students’ diverse learning needs through strategic content, process and learning environment modifications, flexible grouping, scaffolding, and continuous assessment, all aimed at enhancing writing skills in a supportive and inclusive classroom environment.

Assessment was ongoing and formative, utilizing various methods such as classroom observations, review of students’ writing tasks, one-on-one discussions, and writing tests. The assessment criteria were outlined in a rubric that focused on content, grammar, vocabulary, mechanics, and organization of ideas. This comprehensive evaluation helped monitor individual progress and guided instructional adjustments. To determine participants’ achievement levels, we did not rely on a single test score. Instead, we employed a combination of assessments, including student’ previous semester writing performance, the teacher’s judgement about the students’ writing performance and observation

### 3.8 Methods of data analysis

Data from classroom observation and interview was analyzed, synthesized and meaning is made out of it by using the appropriate method of data analysis in qualitative research [41]. The analytical procedures employed in this study align with the frameworks outlined by [36] and [42] incorporating steps such as organizing & familiarizing, coding & reducing, interpreting & representing as in [36] and data managing, reading & memoing, describing, classifying, interpreting, representing and visualizing as in [42]. By systematically organizing, coding, and interpreting the data, the study aimed to identify significant themes and patterns, and to provide a nuanced understanding of the participants’ experiences within the educational context, ultimately enhancing the validity and reliability of the findings.

### 3.9 Validity and reliability of the study

To enhance the reliability of the study, the researchers practiced reflexivity by maintaining a reflective journal and engaging in peer debriefing throughout the research process. Methodological triangulation was employed by combining interviews with students and their teachers, along with classroom observations, to cross-verify findings and enrich the data. While both methods aimed to collect rich and in-depth information, there remains a possibility of social desirability bias influencing participant responses during interviews. To mitigate this, measures such as guaranteeing anonymity and creating a supportive environment were implemented; however, some degree of bias may still persist. These techniques, alongside expert reviews and comments, were employed to strengthen the overall reliability and validity of the findings, ensuring a thorough and credible research process.

## 4 Results and discussions

To thoroughly examine the results and their implications, it is important to first grasp the demographic characteristics of the study’s participants. Table 1 above, presents key information about the students’ backgrounds, offering insights into the diversity of the student population and aiding in the contextualization of the findings.

**Table 1:** Students’ Demography.

Demographic Variable	Details (n = 32)
Gender	
Female	20
Male	12
Age Range	12-13
Average Age	12.5

Table 1 presents the demographic characteristics of the study participants, consisting of 32 students that fall within the age range of 12–13 years, with an average age of 12.5. Given that the study focuses on students’ experiences with DI as a teaching approach to writing, the relatively homogenous age group suggests a specific developmental perspective on the effectiveness and reception of such instructional methods. This demographic context is essential for interpreting the findings, as it helps to contextualize how different genders and a precise age range may influence students’ experiences and responses to DI in writing.

Table 2 above, displays demographic data of the participant teacher, a 45-year-old male with 20 years of experience and a first-degree holder in teaching the English language, demonstrates a wealth of expertise in teaching. His extensive expertise in teaching English provides valuable insights into the DI approach.

**Table 2:** Teacher’s Demographic Information.

Demographic variable	Details (n = 1)
Age	45 years
Gender	Male
Years of Experience	20 years
Highest Qualification	First degree in Teaching English

### 4.1 Results of classroom observations

Participation was notably vivid in the classroom, as the nature of the tasks themselves encouraged active engagement and left no room for idleness. With tasks catering to diverse ability levels, all students were observed actively engaged in the provided activities. The teacher’s close monitoring and guidance were evident, fostering a comfortable environment where students felt free to ask questions without fear. The classroom atmosphere felt stress-free; researchers observed that students were not afraid to express their opinions, whether asking questions of others or providing answers when prompted.

Heightened student motivation was also observed. Whenever the teacher introduced a new activity, students listened with eagerness. When grouped, they actively followed instructions and engaged in the activities willingly. The observed atmosphere was lively, with every student having a task to engage with.

The teacher was observed regularly employing various DI strategies (flexible grouping, scaffolding, mini-lessons...etc) and grouping configurations, enabling students to rotate their desks and face each other. Despite some resource limitations—such as markers and chart papers not being available for posting vocabulary lists—the teacher effectively utilized the backs of students’ exercise books for writing vocabulary lists, supporting comprehension and providing essential words for writing tasks.

Interaction was also evident between the teacher and students, as well as among students. As the teacher regularly placed students in different grouping configurations, they participated in their assigned groups with enthusiasm, demonstrated by their full engagement and contribution to the task. The mini-lessons designed together with textbook provided activities provided students with the opportunity to begin at their current level of capability and receive tailored support. Students at the lower ability level were observed working on the mini-lessons, which were designed to address gaps in prerequisite knowledge. Some students were also heard reporting their efforts in reading the mini-lessons at home and addressing their knowledge gaps through self-study.

Similarly, students at the highest level of performance also had activities that were not merely redundant but offered a suitable challenge to their ability level. These students, though few in number, demonstrated genuine eagerness to tackle challenges designed to match their ability level, showing full commitment. The middle level students were also working on tasks at their ability level, and this promoted on-task behaviour. On-task behaviour was the norm, manifested through students’ active participation in asking and answering questions, engaging in class discussions, and offering opinions.

While the DI approach proved effective in fostering student engagement and motivation, a few students were observed struggling with participation and completing homework assignments. This sometimes led to conflicts with the teacher. Nonetheless, the overall findings indicate that the DI approach successfully created an inclusive classroom environment where students at varying ability levels could thrive.

Generally, the DI approach was observed to be effective in enhancing students’ engagement with the lessons. The support provided through the approach, either through the teacher’s follow-up and regular interaction with students or through the design of materials that provided accessible tasks for every student, contributed to evident student participation and motivation.

### 4.2 Results of students’ interview

As shown in Table 3 above, the themes identified from the students’ lived experiences are presented below.

**Table 3:** The formulated theme and central ideas on the lived experiences of Grade 7 Students in DI Classroom.

R.N	Themes	Central Ideas
4.2.1	Tailored Support	Students received support that matched their need.
4.2.2	Peer learning and collaboration opportunities	Students gained opportunities to work with students of varying readiness levels.
4.2.3	Encouraging & supportive learning environment	Students expressed gaining support and encouragement from their teacher, teaching material and their classmates.
4.2.4	Unfamiliarity with the teaching approach	Initially, the students were unclear about the expectations, leading to some disruptions.
4.2.5	Lack of confidence	Some students demonstrated self-doubt and fear of failure, affecting their collaboration.
4.2,6	Lack of collaboration	Some students felt frustrated and resistant to sharing ideas and completing tasks.

#### 4.2.1. Tailored support.

Students shared positive experiences regarding the tailored support they received through differentiated instruction in writing. One student, S1, remarked, “I don’t sit idle; I am given additional activities whenever I finish the given task earlier, and this motivated me a lot,” highlighting how additional tasks keep students engaged and motivated. S3 noted, “The lists of vocabularies and sentence starters that were provided for us helped me a lot as a reference source to look back whenever I need support,” emphasizing the importance of resources that aid students in their writing process. S4 added, “The teacher was always around to give me the support I need; I was so confident due to this,” reflecting how readily available support boosts students’ confidence in their writing abilities. Together, these comments illustrate how tailored support not only fosters engagement but also provides essential resources and encouragement that enhance students’ writing skills.

#### 4.2.2 Peer learning and collaboration opportunities.

The students reported that peer learning and collaboration opportunities significantly enhanced their learning experience by allowing them to work with peers of varying readiness levels. They expressed feelings of comfort and support while collaborating with one another, which facilitated open sharing of ideas and insights. S5 described this dynamic by stating, “With some students, the idea that I am struggling with was at the tip of their tongues, and I found that to be inspirational to work more. It also helped me to grasp things easily and accomplish tasks earlier.” S3 echoed this sentiment, noting that “working in small groups with different students at different times was so motivating because it gave me a chance to learn from different students,” a contrast to their previous learning experiences. S4 added that “when you work with your friends, it is easier to ask questions and discuss because, as peers, you don’t have any reservation to open up yourself.” Furthermore, S4 mentioned, “In small groups, we were not timid to ask questions and make mistakes because our audience was very small in number, and there was no fear of judgment.” Collectively, these insights illustrate how collaborative learning environments foster confidence, inspiration, and a supportive atmosphere for academic growth.

#### 4.2.3 Encouraging & supportive learning environment.

Students expressed that they benefited significantly from an encouraging and supportive learning environment fostered by their teacher, teaching materials, and peers. All the interviewed students highlighted the positive impact of the DI approach in this regard. For instance, S2 stated, “I feel that there is something to get support from—the teacher was there nearby, moving around and watching us do activities. The teaching material also has additional support notes I can refer back to when I feel like using them, and I was also given a chance to work with students who are more able than me, which was a support for me.” S3 supported this idea, noting that the classroom felt more engaging and supportive due to the diverse sources of assistance available. S3 remarked, “The activities in the teaching material were so supportive; I enjoyed the lists of vocabularies provided, the sentence starters, and the revision notes given to me to refer back to them to refresh my memory and get assistance with the tasks I am about to work on.” Together, these reflections underscore how a well-structured, supportive learning environment can enhance students’ confidence and engagement in their educational journey.

#### 4.2.4 Unfamiliarity with the teaching approach..

In the interviews conducted with students who experienced the DI approach, a common theme of initial confusion emerged. S1 expressed, “I was so confused at first because when I was put with different students, it was not easy to mingle and collaborate with them easily.” Similarly, S3 expressed a similar view, stating, “It was not easy for me to refer to the aids in the teaching material because I was not familiar with such a kind of lesson presentation at first.” However, as time progressed, both students noted a positive shift in their understanding and comfort with the approach. S3 proceeded, “But when time went by, I became more familiar with the approach, and I knew where to go when I needed support,” highlighting an important transition from uncertainty to confidence in their learning environment.

#### 4.2.5 Lack of confidence.

The theme of lack of confidence emerged strongly when students reflected on their experiences with the differentiated instruction approach and its various group configurations. Some respondents expressed feelings of resentment toward being grouped with unfamiliar classmates. S4 articulated this idea, stating, “It was not easy to work with students I had not even greeted before. I used to lose confidence in opening up and contributing what I have to say.” This sense of uneasiness was expressed by S6, who noted, “I was not that confident to collaborate with students whom I am not familiar with. If it were with my friends or the people sitting by my side, it would have been much easier to work together, but sitting with various students at different times robs your confidence, I felt.” These reflections underscore how the unfamiliarity with peers can significantly impact students’ willingness to engage and contribute in collaborative settings.

#### 4.2.6 Lack of collaboration.

The theme of lack of collaboration was evident in the frustrations expressed by some students regarding their experiences with the differentiated instruction approach. Some felt resistant to engaging in group activities, leading to a reluctance to share their ideas and contribute to collaborative tasks. S2 articulated this frustration, saying, “It’s hard to feel motivated to work with others when it seems like no one is on the same page. I often found myself doing much of the work alone, without other team members participating.” S6 conveyed her thought, sharing, “I just didn’t want to be called on to share my thoughts because I felt that my ideas wouldn’t be valued. This made me less committed to completing the assigned tasks.”

Moreover, S1 pointed out, “Even if we are in a small group, if all of us do not contribute, then it is meaningless. I have seen some students who are simply idle and have nothing to contribute. This caused frustration for me.” Such comments illustrate the barriers to effective collaboration that can arise when students feel disconnected from their peers or doubt the value of their contributions. When students notice that others aren’t interested or involved, it can make them feel more frustrated and disappointed. This can make it harder for everyone to learn together.

### 4.3 Analysis of teacher’s interview

As shown in Table 4 above, the themes identified from the teacher’s lived experiences are presented below.

**Table 4:** The formulated theme and central ideas on the lived experiences of an English language teacher implementing DI.

S.N	Themes	Central Ideas
4.3.1	Teacher’s perception towards DI strategies as didactic tools in teaching writing	The teacher mentioned that he has a positive perception towards DI except some challenges it posed.
4.3.2	Merits of DI: Increased motivation and participation of students	Due to the tailored support they gained, previously reserved and struggling students became more motivated and increased participation. It brings a learning opportunity for every learner.
4.3.3	Demanding nature of the approach (Disablers)	Initially, the teacher became overwhelmed due to the different roles he had to play in the classroom and the varying level of support he had to give to the students at varying levels. The teacher highlighted lack of resources to implement the approach: time constraint, limited educational resources, large class sizes, lack of teacher training and support, students’ poor language competence, centralized curriculum.
4.3.4	Need collaboration and support from stakeholders (Enablers)	The teacher expressed the necessity of collaboration among parents, students, and the school administration, willingness from teachers’ side, availability of educational resources, support from government side, professional development opportunities for teachers

#### 4.3.1 Teacher’s perception towards DI strategies as didactic tools in teaching writing.

The teacher expressed his positive perception towards the DI approach as a valuable tool in teaching writing. He mentioned that DI is highly effective in engaging every student and ensuring that no one is left unlearned. The strategy also promotes collaboration and trust among students, fostering a sense of community and inclusivity.

From a teacher’s perspective, DI provides immense satisfaction, as it allows him to witness students actively learning and becoming motivated. This, in turn, boosts the teacher’s confidence and motivation to continue implementing the approach. To use the teacher’s word, he said that “I have no reservations whatsoever about the effectiveness of this approach in supporting students’ learning; however, it does require a significant amount of work from the teacher’s side, which, unfortunately, is not readily available in my current context.”

Nonetheless, the teacher remained optimistic, stating that with sufficient time, support from school principals, and other stakeholders, he is confident that the DI approach can elevate education to the next level, bringing about significant improvements in student learning outcomes.

#### 4.3.2 Merits of DI: Increased motivation and participation of students.

The participant teacher was convinced in the transforming impact of DI as a teaching approach. What he has witnessed is students’ increased motivation and engagement. The teacher noted that when tailored attention and resources were provided, students who had once been hesitant to engage in class began to exhibit a newfound enthusiasm for learning. The specific strategies implemented—such as flexible grouping, one-on-one mentoring, and targeted feedback—helped to create a more inclusive and supportive classroom environment. Students who had previously avoided participation showed more comfort in sharing ideas and asking questions, leading to a significant boost in their confidence levels. The teacher observed that this shift not only enhanced individual engagement but also fostered a collaborative spirit within the classroom, as students began to encourage and inspire one another. The teacher also mentioned that if students have a strong foundation of knowledge, this approach can greatly enhance their learning. However, writing skills involve many sub-skills that our students need to develop further in order to succeed. Overall, the tailored support not only mitigated feelings of inadequacy but also ignited a passion for learning, resulting in a more vibrant and participatory classroom dynamic.

Another advantage of differentiated instruction is that it also serves as a motivating factor for the teacher, who finds it rewarding to see students experiencing success in their learning.

#### 4.3.3 Demanding nature of the approach (Disablers)..

The theme of the demanding nature of DI emerged clearly from the teacher’s experiences. Initially, he felt overwhelmed by the many roles required to effectively implement this instructional strategy. Balancing his responsibilities as a facilitator, mentor, and content expert proved to be challenging, and providing personalized support to students with varying skills and needs was particularly taxing.

This multifaceted approach required a deep understanding of each student’s unique learning profile, complicating classroom management further. The teacher noted that the different levels of support needed not only posed management challenges but also demanded emotional awareness. This awareness was essential in order to support students who were struggling to adapt to the new approach and to keep them on track.

This realization led him to reflect on the importance of professional development and additional resources to help educators effectively navigate the complexities of differentiated instruction. Ultimately, his insights revealed a critical tension in this approach: while it has the potential to foster individualized learning, the initial demands placed on educators can be substantial. He emphasized the need for ongoing support and collaboration within the teaching community.

The teacher also mentioned that time constraints and class size posed significant challenges. He stated, “Given the number of students we have and the demanding nature of this approach, I don’t believe it can be implemented easily. If only the school administration would consider reducing class sizes, allocating planning time for teachers, and providing professional development support, this approach could be successfully implemented. Otherwise, it becomes overly demanding and may be more easily ignored than attempted.” He added that this approach requires you to know your students very well so that you can tailor your instruction based on your knowledge of students, but the number you have in one classroom together with other responsibilities you are shouldering do not allow you to go this far in your teaching.

Additionally, the availability of appropriate teaching materials was highlighted as critical; having access to varied resources can greatly enhance the learning experience, allowing for hands-on activities and engaging content that resonates with different students. These contextual factors influence how differentiated instruction works in practice. They show that while this approach has a lot of potential, its success depends heavily on the available resources and structural conditions within schools.

#### 4.3.4 Need collaboration and support from stakeholders (Enablers)..

The interviewed teacher emphasized that collaboration among stakeholders is crucial for the effective implementation of differentiated instruction. He recognized that fostering a conducive educational environment requires a collective effort that extends beyond the classroom. Engaging parents as partners in the educational process is essential. When parents are informed and involved, they can reinforce learning at home and provide valuable insights into their child’s strengths and challenges, thereby creating a supportive environment that aligns with the strategies used in class.

The teacher also highlighted the importance of student engagement. By encouraging students to take an active role in setting their learning goals and reflecting on their progress, educators can foster a sense of ownership and motivation. This approach ensures open lines of communication, cultivating a culture where feedback is welcomed, and students feel empowered to express their needs and preferences.

Moreover, the teacher pointed out the vital role of school administration in facilitating effective differentiation. Administrative support can manifest in several ways, including offering professional development opportunities, allocating necessary resources, and advocating for manageable class sizes. When school leaders prioritize collaboration and allocate resources effectively, they establish a framework in which teachers can thrive and implement differentiated approaches successfully.

In summary, the teacher’s insights illustrate that meaningful collaboration among parents, students, and administration is not merely beneficial but essential for the successful application of differentiated instruction. This collaboration forms a cohesive support system that enhances the educational experience for all students and ultimately leads to improved learning outcomes.

## 5. Discussions and interpretations of the results

The findings of this study indicate that students perceived DI as a beneficial approach for gaining tailored support, motivation, collaboration, and engagement in learning. The students’ reflections highlighted that DI promoted peer learning & collaboration and fostered encouraging and supportive learning environment. Despite initial challenges related to students’ unfamiliarity with the approach and some students’ lack of commitment to assignments, DI was generally perceived as a helpful method for learning writing. This finding is consistent with a previous study [13], which also reported that DI positively impacts students’ writing skills, corroborating the results of the present study. Further support comes from [17] whose work on how DI supports student learning through enhanced engagement, enjoyment, and empowerment is consistent with our observations and student reports.

The study also found that DI accommodates different readiness levels, enhances learning achievement, reshapes students’ attitudes towards learning English, and fosters a collaborative learning environment. [14] similarly supports the effectiveness of DI in fostering English language learning and improving writing competence, respectively. Our study’s results are also consistent with other findings from [15] and [16] which demonstrated the positive impact of DI on students’ attitudes and perceptions in educational settings.

The analysis of students’ readiness levels revealed notable variations in their responses to DI strategies. Classroom observations and interviews indicated that students at higher readiness levels exhibited greater engagement and independence in their writing tasks, while students at lower readiness levels faced more difficulties in applying the strategies. These findings suggest that students’ readiness significantly influences their ability to benefit from DI, emphasizing the importance of tailoring instructional approaches to meet diverse needs. For example, high-achieving students showed enthusiasm for the activities and strategies employed, and they demonstrated interest in the challenges provided. Students at the medium performance level appreciated the additional tailored support, which appeared to enhance their confidence and participation. Conversely, low-achieving students often displayed confusion and lacked confidence in collaborating with peers. This varied response to DI based on readiness levels can be attributed to individual differences among students. Overall, despite these differences, all students exhibited a positive outlook toward the approach. To maximize its effectiveness, students across all readiness levels require more tailored support and sufficient time to familiarize themselves with the strategies, enabling them to fully benefit from differentiated instruction. The favourable student response to DI underscores the importance of incorporating student viewpoints in instructional design. Given that students’ attitudes play a crucial role in shaping their learning experiences [18] and [19], educators should prioritize implementing DI strategies that align well with student preferences.

The teacher’s perception of DI as a pedagogical tool was also positive. He fully embraced the facilitating nature of the DI approach for teaching students at different readiness levels, strongly believing that DI offered a solution for meeting and supporting diverse student needs. The teacher’s primary reservation concerned the required resources and support for full implementation. He suggested that if teachers were provided with the necessary facilities and support, DI strategies could be highly effective in addressing quality issues in education. The study’s findings, particularly from the teacher interview, also indicated the need for continuous support and professional development opportunities for teachers to facilitate effective DI implementation. This aligns with [27] assertion that teachers recognize the benefits of DI and [22] confirmation of teachers’ highly positive perceptions regarding DI’s impact on engagement, participation, learning, and motivation. The identified need for professional development support is consistent with [28] findings, which also highlight its importance as a requirement for DI implementation, and is further supported by local research by [23], which concluded that trained teachers hold positive perceptions regarding DI’s relevance.

Another significant finding from this study identifies both disabling and enabling factors for the implementation of DI. Key disablers include contextual realities such as large class sizes and time constraints that hinder effective planning, in addition to teachers’ unfamiliarity with the DI approach and limited educational resources. Conversely, teachers’ experience and qualifications, availability of professional development opportunities, and collaboration from stakeholders such as parents, students, and school administration were identified as enablers for the fuller realization of DI. These findings resonate with previous research [5,16,18,21,23–25,27] and [26], which similarly highlight the significance of contextual and collaborative elements in successfully adopting DI pedagogies. Overall, the findings underscore the need for supportive systems and resources to enhance the effective adoption of differentiated approaches in diverse classroom environments.

The interplay between these factors highlights the complex challenges and opportunities that teachers face in implementing DI. While systemic issues like overcrowded classrooms and limited resources pose significant challenges, the presence of different student needs and supportive government policies can motivate and empower educators to adopt differentiated strategies. Addressing the disablers and leveraging the enablers will be crucial in creating a more inclusive and effective educational landscape for all students, one where every student can thrive.

Incorporating DI into English language classrooms in Ethiopia holds significant potential for enhancing the quality of education. Implementing DI strategies early on may help tackle persistent challenges within the education system, especially those related to student engagement and achievement. By prioritizing DI and actively seeking alignment between instructional practices and student preferences, educators can create engaging learning experiences that maximize student engagement and achievement, fostering an environment conducive to effective learning and academic success in English language education.

## 6. Conclusions

The findings of this study lead to several key conclusions regarding the implementation of DI in English language classrooms. Students’ engagement with DI reflected a positive perception, significantly enhancing their academic achievement, motivation, collaboration, and overall learning experiences. This suggests that incorporating DI strategies not only nurtures a supportive learning environment but also addresses the diverse needs and readiness levels of students, leading to improved engagement and outcomes.

Furthermore, the favourable attitudes of students toward DI underscore the importance of adapting instructional methods to meet their preferences, thereby facilitating better learning experiences. Given that these positive perceptions are linked to noticeable benefits in academic performance, it is essential for educators to move away from traditional, one-size-fits-all teaching approaches in favour of more responsive strategies that recognize individual differences among learners.

The evidence presented reinforces the call for stakeholders to prioritize professional development and resource allocation that support the effective implementation of DI in the Ethiopian educational context. By equipping teachers with the necessary skills and knowledge, sxthe quality of English language instruction can be significantly enhanced, addressing persistent challenges in student engagement and achievement. This study not only adds to the existing literature on DI, particularly within the Ethiopian context, but also serves as a practical guide for educators seeking to implement differentiated strategies effectively. By building on prior research showing DI’s benefits, this work lays a foundation for future investigations into its applicability across various language skills, grade levels, and educational contexts. Overall, the integration of DI in EFL classrooms holds the potential to transform English language teaching, fostering an inclusive and enriching educational experience for all students. Top of Form

## 7. Limitations and future directions

This qualitative study, employing classroom observations and interviews, has limitations that may impact the findings. Firstly, researcher bias is a significant concern, as our interpretations might be influenced by personal beliefs or expectations about DI. To mitigate this, we practiced reflexivity by maintaining a reflective journal and engaged in peer debriefing. This helped us monitor our biases and ensured a more objective analysis of the data.

Secondly, while both methods aimed to collect rich data, there is a possibility of social desirability bias affecting participant responses during interviews. We addressed this by guaranteeing anonymity and fostering a supportive environment, but some level of bias may still be present. Additionally, the small sample size and specific context limit the generalizability of our findings. While in-depth insights were gained from grade seven students and their English language teacher, these may not represent all educational contexts. Future research should include a more diverse participant pool to enhance applicability.

Lastly, focusing on specific classrooms may overlook broader systemic factors impacting DI. We recommend future studies consider a multi-site approach to gain a more comprehensive understanding of DI across various educational settings. By recognizing these limitations, we hope to guide future research in further exploring the complexities of DI.

## Supporting information

S1 Appendix Transcripts of Student Interviews. (DOCX)

S2 Appendix Transcripts of Teacher Interviews. (DOCX)

S3 Appendix Classroom Observation Checklist. (DOCX)

S4 Appendix Teacher Interview Protocol. (DOCX)

S5 Appendix Student Interview Protocol. (DOCX)

S6 Appendix Summary of data analysis procedure. (DOCX)
