# Supplementary material for: A phenomenological study of differentiated instruction experience in an Ethiopian middle school: The case of grade 7 students in Hawssa city, Ethiopia
Source: PLoS One. 2026 Jan 16;21(1):e0341025. doi: 10.1371/journal.pone.0341025 (PMC12810785; doi:10.1371/journal.pone.0341025)
Supplement: S1 Appendix — (DOCX) [file pone.0341025.s001.docx]

**S1 Appendix: Transcripts of Students’ Interview**

**Student 1(S1)**

1. **Did you enjoy the writing activities you’ve been doing with the new method?
   If yes, what did you like most?**

**If not, what didn’t you like?**

**S1:** Yes, I enjoyed the writing activities we have been doing through differentiated instruction. It was full of supporting elements. Our teacher was also closer to us better than other times. The support I gained from my teacher and classmates has also helped me to view the new teaching method positively.

1. **Did you feel you were actively participating in the writing activities provided?**

**S1:** Yes, I was participating highly. There were varieties of tasks for me to engage in. I didn’t sit idle; I am given additional activities whenever I finish the given task earlier, and this motivated me a lot. When we were working in groups also, I participated in the activities given.

1. **How do you express your motivation towards the writing activities provided to you?**

**S1:** I was highly motivated with the nature of the tasks themselves. For me, there was always something new to expect, and this motivated me a lot. When I was done with the task in my hand, I was not made sit idle, there was additional task to keep me engaged. Additionally, I was also given a chance to write on the topic of my interest which I found to be very motivating for me.

1. **Did you feel you got the support you need during the writing classes conducted through the new approach to teaching writing?**

**S1:** Yes. There was a lot to support us. Lists of vocabularies, sentence starters and the peer support we have been getting are the major ones.

1. **Can you compare the way you have been learning writing through the new approach with the one you had been learning writing before? (In terms of student grouping, participation, support given, additional activities provided.) What differences have you observed?**

**S1:** When I compare the new approach with the previous teaching method, I have seen differences.

**Q. In terms of students grouping?**

**S1:** Student grouping was not that much practiced in the previous teaching method. Even if there is grouping, it was not similar with the one in the new method. What has been done in the new method was making students work with different students (by mixing them from various sitting areas). In the previous method, students grouping was practiced rarely and when it is done, it is between or among students who are sitting near each other.

**Q. In terms of participation?**

**S1:** In terms of participation, I believe the new approach gives more chance for participation but I didn’t see participation being done uniformly across whole class. In the new approach, we used to sit in small group with our classmates but even if we are in a small group, if all of us do not contribute, then it is meaningless. I have seen some students who are simply idle and have nothing to contribute. This caused frustration for me.

**Q. In terms of support and additional activities provided?**

**S1:** The new method was full of support from the teacher, the teaching material, the classmates and additional tasks (home taken) tasks provided. Such things were not that much visible in the old method of teaching.

**Q. Which ones do you suggest to be incorporated in your future learning of writing English? Which one do you suggest not to appear in your future learning?**

**S1:** I suggest integrating the new approach into the previous teaching method. The new approach has lots of positive sides but again it needs some considerations. For example, working with students you are not familiar with was not easy. From my experience, I was so confused at first because when I was put with different students, it was not easy to mingle and collaborate with them easily. So, it takes time to teach students what to do in the new approach and it needs support from the teacher.

**Student 2 (S2)**

1. **Did you enjoy the writing activities you’ve been doing with the new method?**
   **If yes, what did you like most?**

**If not, what didn’t you like?**

**S2:** I enjoyed the writing lessons with the new method of teaching. It was full of support and guidance and there were also activities to choose from. I also liked the way we have been put with different students to work with.

1. **Did you feel you were actively participating in the writing activities provided?**

**S2**: Yes. I was participating on the tasks I was given to accomplish. Even when I feel the task is hard to accomplish, as there were supporting elements I feel confident to try. We were also supporting each other and working together with the other group members, except for lack of commitment from some group mates.

**Q**. Can you explain it more?

**S2:** I liked many of the works we have been doing but there were few areas that I felt bad about. Say for example, not all the group members were contributing to the given task sometimes. Some students simply sit idle without making contribution to the task, they sometimes do not do given home works and I found this to be frustrating. It’s hard to feel motivated to work with others when it seems like no one is on the same page. I often found myself doing much of the work alone, without other team members participating. Apart from this I found the new approach to be very interesting.

1. **How do you express your motivation towards the writing activities provided to you?**

**S2:** I found it to be very motivating. There is something interesting in this approach…like the grouping, the support on the teaching material…. the teacher’s Prescence. we even used to change seats …which I found to be so motivating. There was a chance to work with various students and it makes the lesson interesting.

1. **Did you feel you got the support you need during the writing classes conducted through the new approach to teaching writing?**

**S2:** Yes! I feel that there is something to get support from—the teacher was there nearby, moving around and watching us do activities. The teaching material also has additional support notes I can refer back to when I feel like using them, and I was also given a chance to work with students who are more able than me, which was a support for me. Our teacher used to give us lists of vocabularies to chose from, lists of sentence joining words, lists of sentence starters and also, he used to help us generate ideas by drawing pictures.

1. **Can you compare the way you have been learning writing through the new approach with the one you had been learning writing before? (In terms of student grouping, participation, support given, additional activities provided.) What differences have you observed?**

**S2:** Can you repeat your question please?

**Q: yes. Can you compare the new approach and the previous one in terms of students grouping, say for example?**

**S2:** yes. Grouping was highly practiced in the new approach. We used to put in groups in the previous approach too but the group members in the new approach always change. Sometimes you work with the students sitting by your side, sometimes you change your seat to work with other students may be sitting at the back or the front of the class. And you also get a chance to work with classmates who are more able than yourself and sometimes with students who are less performing than you. In the previous method of teaching there is no such a thing.

**Q. In terms of participation?**

**S2:** The same is true in terms of participation too. As there were lots of support in the new approach, our participation also increased. In the previous method, few able students will participate but the majority of us sit idle. In the new method, almost many of us were given opportunities to participate.

**Q. In terms of support and additional activities provided?**

**S2:** As I have mentioned it before, the new approach has better support for students than the previous teaching method. There were also additional activities for us to choose from in the new approach which was not available in the previous teaching method.

1. **Which ones do you suggest to be incorporated in your future learning of writing English? Which one do you suggest not to appear in your future learning?**

I want the new approach to be a teaching approach in the learning of writing for the future.

**Student 1(S3)**

1. **Did you enjoy the writing activities you’ve been doing with the new method?**
   **If yes, what did you like most?**

**If not, what didn’t you like?**

**S3:** I liked the writing lessons I have been learning with the new method of teaching. I enjoyed the support I gained in it. The lists of vocabularies and sentence starters that were provided for us helped me a lot as a reference source to look back whenever I need support and this made like the new method very much.

1. **Did you feel you were actively participating in the writing activities provided?**

**S3:** I think so. I was engaging in the tasks I was assigned to and contributing what I have to the group I am part of. Yes, I was participating.

1. **How do you express your motivation towards the writing activities provided to you?**

**S3:** I was so motivated. It was a new experience and it was not boring. working in small groups with different students at different times was so motivating because it gave me a chance to learn from different students. The teachers close follow up as also so motivating.

1. **Did you feel you got the support you need during the writing classes conducted through the new approach to teaching writing?**

**S3:** Support was there. when I struggle in remembering sentence structures or appropriate vocabulary for writing, there were lists in the word bank. I was also given additional reading notes to be taken to home and to read. It was also a support for me. Generally speaking, the activities in the teaching material were so supportive; I enjoyed the lists of vocabularies provided, the sentence starters, and the revision notes given to me to refer back to them to refresh my memory and get assistance with the tasks I am about to work on.

1. **Can you compare the way you have been learning writing through the new approach with the one you had been learning writing before? (In terms of student grouping, participation, support given, additional activities provided.) What differences have you observed?**

**S3:** There are differences between the new and the previous method of teaching. In terms of student grouping, for example, the grouping method used in the new approach was not permanent grouping -there was a chance for every student to work with every student in the class, what was practiced in the previous method of teaching was somehow permanent grouping technique- you work with students around you. I found the grouping method in this approach to be interesting and helpful for my learning.

In terms of participation too, there is difference. In the new approach almost all of us were participating, contributing and it is different from the previous method of teaching in which fewer students were given a chance to participate and chances are limited for the other students who are not sitting in front of the teacher. In terms of support and additional activities provided, the new method was so supportive for the students. You have resources to refer to, your classmates are also here sitting with you working with you, whenever you have question there are students to support, there is the teacher to guide you. So, support was so visible and additional supportive activities are also there to study. The previous teaching method was not like this.

**Q. Which ones do you suggest to be incorporated in your future learning of writing English? Which one do you suggest not to appear in your future learning?**

**S3:** I suggest the new approach to be the teaching method for writing and other subjects too. I want it to be the usual teaching method in English classes and other subject areas. When is it made the common practice, the confusion I had with the new approach will be eliminated.

**Q: What do you mean by the previous confusion you had?**

**S3:** When I say the previous confusion, I am referring to the challenge I had with the new approach during the first one or classes. During these times, it was not easy for me to refer to the aids in the teaching material because I was not familiar with such a kind of lesson presentation at first. But when time went by, I became more familiar with the approach, and I knew where to go when I needed support. So, when we become more familiar with the approach, I believe, this method will benefit us a lot.

**Student 4 (S4)**

1. **Did you enjoy the writing activities you’ve been doing with the new method?
   If yes, what did you like most?**

**If not, what didn’t you like?**

**S4:** Yes, I enjoyed it somehow but not fully. The support I gained from my teacher was great. There were also students who were so supportive but you know…you might not get along with all your group mates. To begin with we were not familiar with such teaching method and I think it needs time to familiarize yourself with all the techniques used in it. For me, it was not easy to work with students I had not even greeted before. I used to lose confidence in opening up and contributing what I have to say.

1. **Did you feel you were actively participating in the writing activities provided?**

**S4:** Yes, I believe I have been participating well in the activities given. Because were working in small groups, it was easier for me to participate. when you work with your friends, it is easier to ask questions and discuss because, as peers, you don’t have any reservation to open up yourself. In small groups, we were not timid to ask questions and make mistakes because our audience was very small in number, and there was no fear of judgment. The challenge I had with this approach is with some students who were not contributing as they are supposed to. Othe than that Participation was great for many of us.

1. **How do you express your motivation towards the writing activities provided to you?**

**S4:** My motivation was great due to the nature of the activities, like I was given a chance to write on a topic that I am interested in, so are the other students and it increased my motivation. The teacher’s close follow up and support was also so motivating. The teacher was always around to give me the support I needed; I was so confident due to this. Because of these factors, I like the new method of teaching and I found it to be so motivating.

1. **Did you feel you got the support you need during the writing classes conducted through the new approach to teaching writing?**

**S4:** I have received lots of supports in the new teaching method. To begin with, the teachers’ support was so helpful for me. The support I gained from the material is also great. The vocabularies we were told to study and use in our writing also helped me a lot. The note I was provided to work on/study also supported me to remember what I have forgotten from my previous studies.

1. **Can you compare the way you have been learning writing through the new approach with the one you had been learning writing before? (In terms of student grouping, participation, support given, additional activities provided.) What differences have you observed?**

**S4:** There are differences

**Q. In terms of students grouping?**

**S4:** In the new method of teaching, group members are not permanent; they change from time to time. You are not made to work with the people you are working with for one activity. Membership changes now and then. In the previous method, the grouping was permanent and it was not implemented frequently.

**Q. In terms of participation?**

**S4:** Participation is better in the new method of teaching. There were chances for every student to participate. There were support systems for everyone to get supported and participated. In the previous method of teaching, participation was something left for few students who are good at their academic performance. The new method encourages every student to contribute what they have.

**Q. In terms of support and additional activities provided?**

**S4:** In terms of support and additional activities, the new method is rich in this regard but the old method lacks these supporting elements in it.

**Q. Which ones do you suggest to be incorporated in your future learning of writing English? Which one do you suggest not to appear in your future learning?**

**S4:** I like the new approach. However, when working with students who are not well familiar with each other, it is important to establish some rules to facilitate effective collaboration and encourage the students to contribute. Additionally, we must ensure that everyone puts in effort and contributes to the assigned work. Aside from these considerations, I find the new method to be very beneficial, and I recommend it as the teaching approach for the future.

**Student 5 (S5)**

1. **Did you enjoy the writing activities you’ve been doing with the new method?**
   **If yes, what did you like most?**

**If not, what didn’t you like?**

**S5:** Yes, I have enjoyed the new method of teaching very much.

**Q: Do you have a reason?**

S5: Yes. I like the collaboration among us and also the support we have gained from the teacher. I was getting better support and guidance from my teacher and it used to boost my confidence to learn more, to ask more.

1. **Did you feel you were actively participating in the writing activities provided?**

**S5**: Yes. I think so. As there were support from everyone -the teacher, groupmates and the teaching material itself-I have tried to participate better than I did in the previous teaching method.

1. **How do you express your motivation towards the writing activities provided to you?**

**S5:** My motivation was high, I think. I loved the activities, the support and collaboration among us and due to this I was eager to come to class and learn new things.

1. **Did you feel you got the support you need during the writing classes conducted through the new approach to teaching writing?**

**S5:** Yes indeed. Support was there from everyone. When I work with students of various academic performance, I got inspired to study more and to make better contribution to my group. With some students, the idea that I am struggling with was at the tip of their tongues, and I found that to be inspirational to work more. It also helped me to grasp things easily and accomplish tasks earlier. The teacher was also so helpful in supporting us while struggling with our writing. Generating ideas was not easy for me, but the teacher often used drawings to illustrate how I could use images to help generate ideas for my writing.

1. **Can you compare the way you have been learning writing through the new approach with the one you had been learning writing before? (In terms of student grouping, participation, support given, additional activities provided.) What differences have you observed?**

**S5:** Yes. The two methods of teaching have differences.

**Q: In terms of grouping?**

**S5:** The new method uses student grouping regularly and there is a chance for us to work with almost everybody in the classroom at various times and it gave us a chance to know each other better and to get support better from more able students. In the old method of teaching, grouping was there, but not frequent and it was done among or between students sitting near each other.

**Q. In terms of participation?**

**S5:** I say participation was better in the new method of teaching because, as I have mentioned it earlier, the new method has lots of support and it makes us feel confident to contribute/ participate in the activities being done. Contrary to this, the previous approach gives chance only for able students and the rest of the class just sits and listens to what the other students say.

**Q. In terms of support and additional activities provided?**

**S5:** I would say the new method is better in this regard as well. It offered additional activities and tasks for us to choose from, which were not available in the previous teaching method.

1. **Which ones do you suggest to be incorporated in your future learning of writing English? Which one do you suggest not to appear in your future learning?**

**S5:** I would suggest the new method to be implemented in the future but with some orientation to some students. You know, some students were not collaborating and contributing as they ought to. Some looked confused and some were even coming to class without completing home taken assignments.

**Student 6 (S6)**

1. **Did you enjoy the writing activities you’ve been doing with the new method?**
   **If yes, what did you like most?**

**If not, what didn’t you like?**

**S6:** I enjoyed it to some degree because of the support it gave me in my writing. It also made me work better. But there were aspects of the new method I was not comfortable with the new method too. When I was made to sit with different students at different times, I was not that confident to collaborate with students whom I am not familiar with. If it were with my friends or the people sitting by my side, it would have been much easier to work together, but sitting with various students at different times robs your confidence, I felt.

1. **Did you feel you were actively participating in the writing activities provided?**

**S6:** To some degree, yes. Especially when I got a chance to work with my friends, I have tried to participate but with new group members, it was not easy for me. I just didn’t want to be called on to share my thoughts because I felt that my ideas wouldn’t be valued. This made me less committed to completing the assigned tasks and participate.

1. **How do you express your motivation towards the writing activities provided to you?**

**S6:** As the new approach has more support from my teacher, it motivated me better.

1. **Did you feel you got the support you need during the writing classes conducted through the new approach to teaching writing?**

**S6:** Yes. Especially the support I gained from my teacher and also from the additional reading materials provided were paramount.

1. **Can you compare the way you have been learning writing through the new approach with the one you had been learning writing before? (In terms of student grouping, participation, support given, additional activities provided.) What differences have you observed?**

**S5:** Yes. They have differences.

**Q: In terms of grouping?**

**S5:** The new method is full of grouping activities but the previous method has few tasks to be done in groups. Again, group members in the new method change regularly, in the previous method members were permanent.

**Q. In terms of participation?**

**S5:** For me participation was better in the previous teaching method, but in the new method, working with students whom I didn’t even greet before was not easy. But I think the other students liked the way they have been working with different students at different times.

**Q. In terms of support and additional activities provided?**

**S5:** I would say the new method is better in this regard as well. It offered additional activities and tasks for us to choose from, which were not available in the previous teaching method. I was not that confident to easily mingle with students I am not familiar with.

1. **Which ones do you suggest to be incorporated in your future learning of writing English? Which one do you suggest not to appear in your future learning?**

**S5:** I suggest that the new approach be adopted as the teaching method for the future, but with some modifications. I appreciate the support it provides, but I would prefer it if we were made to work with students we know well.
