# Supplementary material for: A phenomenological study of differentiated instruction experience in an Ethiopian middle school: The case of grade 7 students in Hawssa city, Ethiopia
Source: PLoS One. 2026 Jan 16;21(1):e0341025. doi: 10.1371/journal.pone.0341025 (PMC12810785; doi:10.1371/journal.pone.0341025)
Supplement: S2 Appendix — (DOCX) [file pone.0341025.s002.docx]

**S2 Appendix: Transcripts of Teacher’s Interview**

**I. Demographic/Background Information**

**Question:** What is your highest level of education?

**Response:** I hold a first degree in English Language Teaching

**Question:** How many years have you been working as a teacher?

**Response:** I have 20 years of teaching experience.

**Question:** Which grades have you taught in the past?

**Response:** I have been teaching primary level students from grade 1 through 8.

**Question:** How long have you been teaching in seventh grade?

**Response:** It is now my sixth year.

**Question:** If you are comfortable, you can also tell me your age.

**Response:** No problem at all. I am 45 years old now.

**II. Experiences with DI Implementation**

1. **How has the Differentiated Instruction approach influenced your instructional practices in teaching writing?**

**Teacher:** Differentiated Instruction has fundamentally influenced my teaching practices in several ways. I have adapted my methods in several key ways to implement DI effectively in the classroom. Firstly, I employ flexible grouping strategies, utilizing whole-class, small-group, and individual configurations based on students’ readiness levels, which promotes both targeted instruction and peer learning. Additionally, I incorporate a variety of instructional methods, including direct instruction, collaborative learning, independent projects, and hands-on activities, to cater to diverse learning preferences. I modify curriculum materials—such as texts, writing prompts, and graphic organizers—to meet different readiness levels, ensuring all students can engage with the content meaningfully. Ongoing assessment and feedback are integral to my approach; I conduct frequent formative assessments to inform my instruction and provide timely feedback that guides student improvement. Moreover, I promote student choice and autonomy by offering a selection of assignments and projects tailored to their strengths, thereby increasing engagement and fostering responsibility for their own learning. I also strive to create a supportive classroom environment that values diversity, setting clear expectations for collaboration and respect, which encourages students to take risks in expressing their ideas. Overall, this approach has made me more flexible and creative in my teaching. I’ve seen improvements in student engagement and confidence in their writing abilities, which is incredibly rewarding as an educator.

1. **What challenges, if any, have you encountered while implementing this approach in the classroom?**

**Teacher:** While Differentiated Instruction offers numerous benefits, I have faced several challenges in implementing this approach in my classroom. I am grateful for the support I received, including tailored teaching materials, training on implementation, and assistance in redesigning instructional activities and resources. Without these supports, success would have been much more difficult. I can only imagine how demanding it would have been to implement Differentiated Instruction without such guidance.

Even with these supports, I still faced obstacles. The greatest challenge was the large class size; managing students and meeting their diverse needs during writing exercises would be highly demanding given the number of students typically present in a regular class. While, in this case, the class consisted of only 32 students—since participation was based on consent, I can imagine how much more challenging it would have been with a larger group. Ensuring that each student received appropriate guidance often felt overwhelming, especially in the initial weeks when I was still gaining confidence and organizing my approach. Honestly, having an assistant teacher would have greatly alleviated some of these pressures and helped better support the students' individual needs.

Another challenge was assessing students' work and providing tailored feedback. With students working on different activities based on their individual writing proficiency levels, offering specific support became demanding. Having little time made the challenges even harder. It requires preparation time from the teacher side to familiarize oneself with the different strategies and choose which one suits which need requires significant time. Additionally, incorporating these activities within the designated curriculum timeline proved to be a challenge.

Student resistance was another obstacle I faced. Some students are not always receptive to differentiated tasks; they may feel uncomfortable with working at different levels or prefer a more traditional, uniform approach. Addressing these mind-sets and helping them understand the value of differentiation has required ongoing effort and communication.

Furthermore, I believe that implementing this approach necessitates more training and support. Although I've endeavoured to apply these strategies, additional professional development focused on differentiated instruction would enhance my ability to effectively meet the diverse needs of my students. Lastly, resource limitations present another challenge. A lack of adequate teaching materials and technology—such as papers, markers, and glue—hinders the full implementation of DI. Often, I am unable to provide a variety of writing tools and resources for students to choose from, which restricts their engagement in different writing activities. Given the number of students we have and the demanding nature of this approach, I don’t believe it can be implemented easily. If only the school administration would consider reducing class sizes, allocating planning time for teachers, and providing professional development support, this approach could be successfully implemented. Otherwise, it becomes overly demanding and may be more easily ignored than attempted.

1. **What supports or enablers have you experienced while implementing this approach?**

Implementing Differentiated Instruction has been significantly facilitated by several supports and enablers within my teaching environment. One of the most impactful resources has been the availability of tailored teaching materials and activities I was provided with. These materials are specifically designed to meet the diverse needs of my students, allowing me to implement DI more effectively. They provide a variety of writing prompts and scaffolding activities that cater to different skill levels, making differentiation more manageable.

Additionally, the professional development opportunities I have received, along with the ongoing follow-up and continuous communication support, have been invaluable. These professional development sessions focused on DI have enhanced my understanding of differentiation principles and equipped me with practical strategies to apply in the classroom. As a result, I feel more confident and prepared to meet the needs of my students.

Another crucial support has been the positive feedback from my students. Their encouraging responses have been a significant motivating factor for me. Many students have expressed appreciation for the flexibility and choices in their writing tasks, inspiring me to continue utilizing DI strategies. Their enthusiasm for engaging with the material reassures me that I am on the right path.

Overall, these supports have played a critical role in my ability to implement Differentiated Instruction effectively, ultimately leading to a more engaging and productive writing experience for my students.

1. **Have you noticed any changes in your students' participation, motivation, or confidence in writing as a result of this approach?**

Yes, I have definitely noticed positive changes in my students' participation, motivation, and confidence in writing as a result of implementing Differentiated Instruction. One of the most significant changes I've observed is a remarkable increase in student engagement during writing activities. By offering diverse prompts and allowing students to select topics that suit their level, I see more students actively participating in class discussions and eagerly starting their writing tasks. This renewed engagement has transformed what was once perceived as a chore into a more enjoyable and meaningful experience.

The tailored support embedded in the teaching materials, which aligns with students' readiness levels, has significantly enhanced their motivation. I’ve noticed that students are utilizing the additional resources available in the materials when needed, which has increased their engagement in the learning process. Even students who previously sat idle are now actively participating and making use of the support provided. As they witness their progress and receive positive feedback on their efforts, their motivation grows, creating a self-reinforcing cycle.

I have also observed greater confidence among my students. In discussing their experiences with them, many have expressed feelings of increased confidence in their writing abilities. As they tackle tasks that match their skill levels, they experience more frequent successes, which boosts their self-esteem. I’ve seen students who were once hesitant or anxious about writing take pride in their work and share it with their peers.

Additionally, the collaborative and supportive culture fostered by DI has positively impacted my students. They are more willing to provide constructive feedback to one another, which not only aids their learning but also builds their confidence in sharing their writing. This supportive environment encourages students to express their ideas more freely.

Finally, the differentiated approach has reduced the frustration that some students previously experienced with writing tasks. By scaffolding assignments and providing varying levels of support, I can see that students feel more capable of tackling the material, leading to a more positive overall experience.

1. **What advantages of using Differentiated Instruction have you experienced? How would you describe these from your students' perspective and from your own perspective?**

From my perspective the enhanced classroom engagement is one of the advantages. Differentiated Instruction has allowed me to create a more engaging classroom environment. By catering to various learning styles and interests, I notice a marked increase in student participation and enthusiasm for writing tasks. It also enables me to tailor my instruction based on the students’ readiness level and this gives me a sense of accomplishment in seeing the students learn something. DI enables me to address the individual needs of my students effectively. It provides a framework to adapt lessons, assessments, and materials, which helps to ensure that all students can access the curriculum at their level. I am also advantageous in that I get a chance for professional growth. Implementing DI has pushed me to explore new teaching methods and continually improve my instructional strategies. This has enhanced my skills and confidence as an educator.

It also helped me in creating a positive classroom atmosphere .The focus on individual strengths and needs promotes a supportive and collaborative classroom atmosphere, fostering a sense of community among students.

From my students' perspective, implementing Differentiated Instruction has led to several significant advantages. First and foremost, many students appreciate having a choice in their learning activities, which fosters a sense of ownership over their education. This autonomy allows them to engage with the material in a way that feels meaningful and relevant to them. Additionally, students often express feeling more successful because tasks are tailored to their individual skill levels. This tailored approach leads to a greater sense of achievement and significantly boosts their confidence in their abilities. Moreover, they enjoy the diverse learning opportunities that DI facilitates, including group work, pair work, and flexible grouping. This variety not only keeps lessons interesting but also motivates them to participate actively in their learning journey. Overall, the implementation of DI has created a more engaging and fulfilling educational experience for my students.

1. **What disadvantages of using Differentiated Instruction have you experienced? How would you describe these from your students' perspective and from your own perspective?**

Implementing Differentiated Instruction has proven to be more challenging than my previous responsibilities. Throughout each class session, I find myself taking on multiple roles, one of which is to monitor progress and ensure that every student stays on task. Additionally, the preparation required to implement the carefully designed teaching materials presents its own set of challenges. With a variety of activities tailored to each student's readiness level, ensuring that the appropriate task is assigned to the right student can be quite difficult. Moreover, learning this new skill can often feel overwhelming and, at times, frustrating. Managing a classroom where students are at different stages and working on diverse tasks adds another layer of complexity. It necessitates constant monitoring and support, which can sometimes lead to time constraints. I have no reservations whatsoever about the effectiveness of this approach in supporting students' learning; however, it does require a significant amount of work from the teacher's side, which, unfortunately, is not readily available in my current context.

From my students' perspective, Differentiated Instruction introduced several challenges. One significant concern was their unfamiliarity with this approach to learning writing. Some students appeared idle and confused when assigned to different group configurations and instructed to engage in activities that differed from those of their classmates. Although they were unaware of which activities were designated for which students, some expressed the feeling that they had received these activities due to a knowledge gap, which disturbed them. However, as time passed, many grew more confident in the tasks assigned and approached them with enthusiasm.

Additionally, in collaborative groups, some students felt excluded or lacked confidence when paired with peers possessing varying skill levels. This dynamic hindered their willingness to participate and share ideas. Moreover, students frequently compared their progress to that of their classmates, leading to feelings of inadequacy if they perceived that they were not advancing as quickly—as DI was designed to support individual growth.

1. **What recommendations would you suggest for implementing Differentiated Instruction in teaching writing during future sessions?**

Implementing Differentiated Instruction in teaching writing can significantly enhance student engagement, comprehension, and skill development. One key recommendation to further incorporate DI into our teaching practice is to provide professional development opportunities for teachers. This professional development should encompass not only the theoretical and practical aspects of DI but also address the attitudes of teachers. From my own experience, I have observed a prevalent misconception among educators—that DI is impossible to implement without sophisticated infrastructure or smaller class sizes—and I too once believed it was solely a one-on-one approach. Therefore, fostering an attitudinal shift is essential.

Teachers need to be equipped with specific strategies designed for effective DI implementation in the classroom. Additionally, I believe that collaboration is crucial. Support from school administration is necessary, as certain materials—such as paper, markers, and chart paper—are essential for adapting teaching resources through the lens of DI and for creating supportive teaching aids.

Moreover, parental awareness of DI is vital; if parents remain uninformed about this approach, they may oppose its implementation, particularly if it involves having students work on content from previous grade levels to address learning gaps. Home assignments also require parental support and collaboration. Students themselves need to understand the goals of DI and be convinced that engaging in diverse activities and additional tasks is beneficial for their learning.

Furthermore, DI should become a cultural norm within the school. Every teacher should embrace this approach to foster collaboration among those teaching the same subject at similar grade levels and across different class levels. It would be advantageous if strategies for DI were included in student textbooks, serving as reminders for teachers during lesson planning and classroom practice.

Lastly, manageable class sizes would greatly benefit DI implementation by enabling teachers to address each student's unique needs. If feasible, providing access to computers would further support students in developing their writing and language skills, enhancing their overall learning experience.
