# Supplementary material for: A phenomenological study of differentiated instruction experience in an Ethiopian middle school: The case of grade 7 students in Hawssa city, Ethiopia
Source: PLoS One. 2026 Jan 16;21(1):e0341025. doi: 10.1371/journal.pone.0341025 (PMC12810785; doi:10.1371/journal.pone.0341025)
Supplement: S3 Appendix — (DOCX) [file pone.0341025.s003.docx]

## S3 Appendix: Classroom observation checklist

## Observation Checklist

**Name of the school: ______________________________________**

**Class size: ___________**

**Observation Number: ______________________**

**Role of Observer: Non-participatory observer**

**Length of observation: -_______________**

| No. | **Parameter** | **Description** |
| --- | --- | --- |
| 1 | Describe the variety of instructional strategies the teacher uses to engage students |  |
| 2 | Describe how the psychological environment of the classroom is conducive for students to express their ideas freely. |  |
| 3 | Provide examples of how the teacher responds to individual student needs during the lesson.( re-teaching, Reinforcing, or extended learning) |  |
| 4 | Describe how students are engaged in tasks, including the types of activities and student participation. |  |
| 5 | How do students engage with the tasks provided—do the tasks seem appropriately challenging, or do they appear bored or overwhelmed? |  |
| 6 | How are different levels of scaffolding reflected in the tasks students are undertaking—do the tasks seem to offer varying support to match students' needs? |  |
| 7 | Describe any informal or formal assessment strategies observed |  |
| 8 | Explain how the teacher gathers and uses assessment information |  |
| 9 | Describe the grouping strategies and their effectiveness. |  |
| 10 | Describe the use and variety of activities for students who finish early. |  |
| 11 | Provide examples of how tasks are tailored for students needing additional help. |  |
| 12 | Description of students’ behaviors during class time, including how they stay on task or become off task. |  |
| 13 | Describe students’ engagement in group work and collaboration with classmates. |  |
| 14 | Describe how students collaborate on writing or other tasks. |  |

Additional Notes:

_______________________________________________________________________________________________________________________________________________________________________________________________________________________________________________________________________________________________________________________________________________________________________________________________________________________________________________________________________________________________________________________________________________________________________________________________________________________________________________________________________________________________________________________________________________________________________________________________________________________________________________________________________________________________________________________________________________________________________________________________________________________________________________________________________________________________________________________________________________________________________________________________________________________________________________________________
