# Supplementary material for: A phenomenological study of differentiated instruction experience in an Ethiopian middle school: The case of grade 7 students in Hawssa city, Ethiopia
Source: PLoS One. 2026 Jan 16;21(1):e0341025. doi: 10.1371/journal.pone.0341025 (PMC12810785; doi:10.1371/journal.pone.0341025)
Supplement: S4 Appendix — (DOCX) [file pone.0341025.s004.docx]

**S4 Appendix: Teacher interview protocol**

Introduction

Thank you for agreeing to participate in this research study and for joining me for this interview today. The purpose of this interview is to gather your insights and experiences regarding the teaching approach—Differentiated Instruction (DI)—in writing, which you have been using for teaching writing. The interview will last approximately 15 to 20 minutes and will cover topics such as your background, your application of DI in writing lessons, the advantages and disadvantages you have observed, and any challenges or supports you have encountered during implementation. I will also ask for your thoughts on next steps for teachers in this area. Please remember that you are not obligated to answer any question, and you may choose to withdraw from the study at any time. As we have agreed in the consent note, the interview will be documented by the interviewer. Do you have any questions before we begin? Your feedback is invaluable to this research and will remain confidential. Thank you for your participation.

I. Demographic/Background Information

1. What is your highest level of education?

2. How many years have you been working as a teacher?

3. Which grades have you taught in the past?

4. How long have you been teaching in seventh grade?

5. If you are comfortable, you can also tell me your age.

II. Experiences with DI Implementation

1. How has the Differentiated Instruction approach influenced your instructional practices in teaching writing?
2. What challenges, if any, have you encountered while implementing this approach in the classroom?
3. What supports or enablers have you experienced while implementing this approach?
4. Have you noticed any changes in your students' participation, motivation, or confidence in writing as a result of this approach?
5. What advantages of using Differentiated Instruction have you experienced? How would you describe these from your students' perspective and from your own perspective?
6. What disadvantages of using Differentiated Instruction have you experienced? How would you describe these from your students' perspective and from your own perspective?
7. What recommendations would you suggest for implementing Differentiated Instruction in teaching writing during future sessions?

Thank you again for your participation. Your insights are greatly appreciated!
