# Supplementary material for: A phenomenological study of differentiated instruction experience in an Ethiopian middle school: The case of grade 7 students in Hawssa city, Ethiopia
Source: PLoS One. 2026 Jan 16;21(1):e0341025. doi: 10.1371/journal.pone.0341025 (PMC12810785; doi:10.1371/journal.pone.0341025)
Supplement: S5 Appendix — (DOCX) [file pone.0341025.s005.docx]

## **S5 Students interview protocol**

Introduction: This interview intends to assess your experiences with differentiated instruction approach to teaching writing. The questions will revolve around your feelings towards the approach and the experience you had with it. Please, feel free to give your opinions briefly and concisely on this interview. The interview will last approximately from 15 to 20 minutes. This interview is only for research purpose. To keep your response confidential, you are not expected to tell me your name.

1. Did you enjoy the writing activities you’ve been doing with the new method?
   If yes, what did you like most?

If not, what didn’t you like?

1. Did you feel you were actively participating in the writing activities provided?
2. How do you express your motivation towards the writing activities provided to you?
3. Did you feel you got the support you need during the writing classes conducted through the new approach to teaching writing?
4. Can you compare the way you have been learning writing through the new approach with the one you had been learning writing before? (In terms of student grouping, participation, support given, additional activities provided.) What differences have you observed?
5. Which ones do you suggest to be incorporated in your future learning of writing English? Which one do you suggest not to appear in your future learning?
