# Supplementary material for: A phenomenological study of differentiated instruction experience in an Ethiopian middle school: The case of grade 7 students in Hawssa city, Ethiopia
Source: PLoS One. 2026 Jan 16;21(1):e0341025. doi: 10.1371/journal.pone.0341025 (PMC12810785; doi:10.1371/journal.pone.0341025)
Supplement: S6 Appendix — (DOCX) [file pone.0341025.s006.docx]

**S6 Appendix: Summary of the data analysis procedure**

**Organizing the data**

Interview data from students and the teacher are transcribed and put in a file for easier access for further analysis.

**Reading and Memoing**

Reading through the entire records from field notes to understand the context and content of the

data. Alongside this, memoing is conducted, during which potential themes are recorded, and

initial broad notes are jotted down.

**Describing, Classifying, and Interpreting Data into Codes and Themes**

At this stage of data analysis, codes are formed either based on what is observed in the data (in

vivo coding) or assigned by the researcher. These codes are then grouped into broader themes,

with similar codes combined under an overarching theme for further interpretation.

**Interpreting the Data**

At this stage of analysis, data interpretation took place which involves researchers ascribing

meaning to the emerging themes by viewing them through existing theories, insights from other

studies, as well as their own personal perspectives and insights.

**Representing and Visualizing the Data**

Data is presented in a tabular form, where themes are listed alongside their central ideas, and these

are discussed in detail.
